# Engineering the Salt-Inducible Ectoine Promoter Region of *Halomonas elongata* for Protein Expression in a Unique Stabilizing Environment

**DOI:** 10.3390/genes9040184

**Published:** 2018-03-28

**Authors:** Lisa M. Stiller, Erwin A. Galinski, Elisabeth M. H. J. Witt

**Affiliations:** Institute of Microbiology and Biotechnology, Rheinische Friedrich-Wilhelms University Bonn, 53115 Bonn, Germany; lisa.stiller@uni-bonn.de

**Keywords:** *Halomonas*, compatible solutes, heterologous expression, salt-induced promoter, ribosome binding site, RBS Calculator, 16S rRNA, GFP, mCherry, ectoine

## Abstract

It has been firmly established that organic osmolytes (compatible solutes) of halophilic *Bacteria* and *Archaea* have positive effects on conformation and activity of proteins, and may therefore improve their functional production. In particular, the amino acid derivative ectoine is known for its conformational stabilization, aggregation suppression, and radical protection properties. The natural producer and industrial production strain *Halomonas elongata* accumulates ectoine in the cytoplasm, and as a result offers a unique stabilizing environment for recombinant proteins. For the construction of broad hoast range vector systems with fluorescent reporter proteins, we chose the salt-inducible promoter region of the ectoine gene cluster (*promA*). A closer inspection of the genetic background revealed that its combination of sigma 38 (σ^38^) and sigma 70 (σ^70^) promoters was followed by a weak ribosomal binding site (RBS). This inspired a systematic approach for the construction of a *promA*-based vector series with a synthetic RBS region using the RBS Calculator v2.0, which resulted in a greatly improved salt-dependent expression—even in a deletion construct lacking the σ^38^ promoter. To expand the application range of this expression system, we looked further into the possible export of recombinant proteins into the periplasm. Both *sec* and *tat* leader sequences from *H. elongata* proved to be suitable for directed periplasmic transport into an extreme environment of freely selectable ionic strength.

## 1. Introduction

The gamma proteobacterium *Halomonas elongata* DSM 2581^T^ (family: *Halomonadaceae*) was first described by Vreeland et al., 1980 [1]. It belongs to a group of versatile halophiles with a broad range of halotolerance and a high degree of adaptability to changing salinity [2,3]. The organism has its growth optimum at a salinity of 2–3% NaCl. Increasing the salinity leads to a reduction in the growth rate (at 10% NaCl reduced by half) and salt-dependent synthesis and/or the accumulation of organic osmolytes, so-called compatible solutes [3]. Its prime compatible solutes responsible for osmotic equilibrium are the tetrahydropyrimidine derivatives l-ectoine and S,S-5-hydroxyectoine. Their biosynthesis and degradation has been investigated in depth [4,5,6,7] and members of the *Halomonadaceae* have since become a popular object for research on osmotic adaptation [8,9,10]. *H. elongata* is currently exploited for the industrial production of ectoine using the “bacterial milking” technique (extraction by osmotic down shock), and subsequent improvements through the genetic engineering of leaky mutants [11,12]. Like many other compatible solutes, ectoines are characterized by their beneficial effect on biomolecules, in particular, proteins, such as conformational stabilization, aggregation suppression, and radical protection properties [12,13,14,15]. Since ectoines, with increasing salinity, amass to molar concentrations within the cytoplasm of *H. elongata* [3], this organism appears to be an ideal candidate for the heterologous expression of delicate proteins in a unique stabilizing environment. The possible advantages for the development of an expression system in a halophile are as follows: (a) due to its broad salt tolerance *H. elongata* can be grown at low salinity (minimum of 1% NaCl), which enables gene transfer by conjugation with non-halophiles, such as *Escherichia coli* [16], (b) by increasing the salinity of the medium the cytoplasmic concentration of the stabilizing solute may be adjusted to any level between 0 and 2 M ectoines, and (c) with the help of leader sequences the desired protein may be exported into a periplasmic environment, which is devoid of compatible solutes due to efficient high affinity uptake systems [17] and exposed to a salt concentration close to that of the medium. In addition, the periplasmic environment can be adjusted, depending on the product´s requirement for stabilization, by changing the type and concentration of inorganic salts of the medium. 

Using fluorescent reporter proteins (both for cytoplasmic and periplasmic expression), we report a systematic and bioinformatic-inspired approach for the optimization of a versatile plasmid-based protein expression system in the “extremophilic” *H. elongata*. This was achieved by investigating, engineering, and adjusting the promoter region of the ectoine gene cluster along with the upstream region of the coding sequence of the target protein.

## 2. Materials and Methods 

### 2.1. Construction of Different H. elongata Strains

*H. elongata* DSM 2581^T^ and *E. coli* S17.1 were obtained from the DSMZ Institute (Braunschweig, Germany). *H. elongata* 2581^T^ appears to be unable to take up free DNA, as required for bacterial transformation [18]. *E. coli* S17.1 possesses the *tra* genes needed for a conjugative transfer of recombinant plasmids into *H. elongata* [19], and was therefore used as a donor for all of the plasmids. For transformation, cells of *E. coli* S17.1 were made competent as described [20,21] and transformed using a simple heat shock protocol. Successfully transformed colonies were selected by growth on chloramphenicol-containing agar plates (‘Antibiotic Broth Medium No. 3’, Oxoid Ltd., Thermo Scientific, Waltham, MA, USA). Conjugation with *H. elongata* DSM 2581^T^ was performed, as described [16] with minor modifications. Prior to the conjugation experiment the main culture of *H. elongata* DSM 2581^T^ was grown to late exponential phase (optical density at 600 nm (OD_600_) of approx. 0.7). Table 1 refers to all strains and plasmids used or created in this work.

### 2.2. Medium, Supplements and Growth Conditions

All of the *H. elongata* strains were grown in minimal medium 63 (MM63), which contains 13.61 g/L KH_2_PO_4_, 4.21 g/L KOH, 1.98 g/L (NH_4_)_2_SO_4_, 0.25 g/L MgSO_4_ × 7 H_2_O, 0.0011 g/L FeSO_4_ × 7 H_2_O, and 5 g/L glucose × H_2_O. pH was adjusted to 7.1 with NaOH. Different NaCl concentrations (% *w/v*) are indicated as suffix following the abbreviation for the medium (e.g., MM63-3%). Chloramphenicol was supplemented in every culture (50 µg/mL) for plasmid maintenance. *H. elongata* strains were cultivated in shaking flasks aerobically at 30 °C and 160 revolutions per minute (rpm). Cell growth was tracked photometrically at 600 nm.

### 2.3. Recombinant Plasmids and Templates

Vector pGFPuv was obtained from Clontech Laboratories, Inc. (Mountain View, CA, USA). Vector pCQ11-ftsZmCh for amplification of mCherry was kindly provided by Fabian Grein, Institute of Pharmaceutical Microbiology, Bonn, Germany [24]. Strain *H. elongata* KB1 [17] was kindly provided by Hans-Jörg Kunte, Federal Institute for Materials Research and Testing, Berlin, Germany.

### 2.4. Vector Construction

For plasmid construction, standard procedures were used together with *E. coli* DH5α. Polymerase chain reaction (PCR) was carried out using *Pfu* polymerase according to its user guide provided by the manufacturer (Pub. No. MAN0012033, Thermo Scientific). Ligation of PCR fragments and/or plasmid backbones was carried out using the T4 DNA Ligase according to its user guide that was provided by the manufacturer (Pub. No. #EL0014, Thermo Scientific).

- *Plasmid pPE, pWUB01 and pWUB02:* The promoter region *promA* upstream of the ectoine biosynthesis cluster was amplified from genomic DNA of *H. elongata* DSM 2581^T^ (pPE) and *H. elongata KB1* (pWUB01), respectively, with primers for_1A and rev_1A (pPE) or rev_2B (pWUB01). For construction of pWUB02 the forward primer was changed to for_2C. Amplified fragments and the destination vector pBBR1-MCS were digested with appropriate restriction enzymes (Table 2) and ligated. Gene *gfp_uv_* was amplified from pGFPuv using primers for_3D and rev_4D, and was ligated into the corresponding vector backbone downstream of the ectoine promoter.
- *Plasmid pSPE1_GFPuv and pSPE2_GFPuv: gfpuv* was amplified from pGFPuv using either primers for_4E and rev_4D (pSPE1_GFPuv) or primers for_5E and rev_4D (pSPE2_GFPuv). Both forward primers for_4E and for_5E comprise a synthetic RBS adapted for expression in *H. elongata* DSM 2581^T^ and *E. coli*, respectively (Table 2). Amplified fragments and the destination vector pPE were digested with appropriate restriction enzymes (Table 2) and ligated.
- *Plasmid pWUB01_sec_mCherry, pWUB01_tat_gfpuv:* Primers for_11H and rev_8H were used to amplify a fragment of *pcoA* encoding the entire signal sequence and the first four amino acid residues of the mature protein, and, in addition, an overlap extension complementary to the *gfpuv* gene. Primers for_13H and rev_4D were used to amplify *gfpuv* gene from pGFPuv without start codon ATG, but an additional overlap extension complementary to the *pcoA* leader. Both of the fragments were fused in an overlap extension polymerase chain reaction using primers for_12H and rev_4D. The resulting product contained PscI and HindIII restriction sites. Primers for_8G and rev_6G were used to amplify a fragment of *teaA* encoding the entire signal sequence and the first four amino acid residues of the mature protein, and, in addition, an overlap extension complementary to the *mCherry* gene. Primers for_10G and rev_7G were used to amplify *mCherry* gene from pCQ11ftsZmCh without start codon but an additional overlap extension complementary to the *teaA* leader. Both of the fragments were fused in an overlap extension polymerase chain reaction using primers for_9G and rev_7G. The resulting product contained PagI and HindIII restriction sites.

### 2.5. Cultivation for Fluorescence Measurements

Precultures of various *H. elongata* strains were prepared in MM63-2% and MM63-6%, and were grown overnight. Main cultures of MM63-2%, MM63-6%, and MM63-10% were inoculated to an OD_600_ of 0.15. Precultures with 2% NaCl were used to inoculate the corresponding main culture and precultures with 6% NaCl were used to inoculate both corresponding MM63-6% and MM63-10% main cultures. All of the cultures were grown to an OD_600_ of approx. 0.9 and harvested. Pellets were stored at −20 °C.

### 2.6. Cultivation for Fluorescence Microscopy

Precultures of various *H. elongata* strains were prepared in MM63-3% and grown overnight. Main cultures in MM63-3% were inoculated with respective precultures to an OD_600_ of about 0.08. At an OD_600_ of 0.4–0.5 half of the main cultures of each strain were supplemented with additional 7% NaCl, resulting in an increased NaCl concentration of 10% in total (further referred to as “salt shock”). Growth was continued in all cultures for about 4 h, and then cultures were used for microscopy. 

### 2.7. Fluorescence Microscopy

For microscopic observation 10 µL of each culture were transferred to a microscope slide with a thin layer of 1% agarose gel (inhibiting cell movement). Microscopy was performed at room temperature with an ‘Axio Observer Z1’ equipped with an Axio Cam MR3 camera. Software: ‘Zen 2’, Zeiss, Oberkochen, Germany. GFPuv excitation and emission: 488/509, mCherry excitation and emission: 587/610. Exposure time for the channels of phase contrast, mCherry fluorescence, and GFPuv fluorescence: 50 ms, 1 s, and 1 s, respectively (except *H. elongata* pWUB_tat_GFPuv in MM63-10%, which was 394.6 ms).

### 2.8. Purification of Soluble Protein Fractions

Cell pellets were thawed on ice and resuspended in 200 µL resuspension buffer (500 mM NaCl, 20 mM Tris-HCl). 2 µL lysozyme (100 mg/mL) were added, reaction tubes were mixed and stored on ice for 30 min. 10 µL of sodium dodecyl sulfate (SDS) (10%) were added, followed by incubation on ice for 30 min. 1 µL of MgCl_2_ (50 mM) and 2 µL of DNAse I (10 mg/mL) were added and the reaction tubes were incubated on ice for 1–2 h, followed by a freezing step (−20 °C) for at least 30 min. The samples were then treated with ultrasound 3 × 15 min in an iced ultrasonic bath. In between sonication steps, the samples were mixed on a vortexer (Labinco B.V., Breda, NL, USA). After centrifugation (15,000× *g*, 4 °C, 30 min), the supernatants containing the soluble protein fractions were transferred into fresh sample tubes and stored at −20 °C.

### 2.9. Protein Quantification

Proteins in the soluble protein fractions were quantified by a bicinchoninic acid assay (BCA assay) with the ‘protein purification kit’, according to the manufacturer's instructions (Interchim Uptima, Montluçon, France).

### 2.10. Polyacrylamide Gel Electrophoresis

Protein samples of soluble protein fractions were diluted with H_2_O_ultrapure_ to a total amount of 20 µg and mixed with 4× reducing sample buffer (45% stacking gel buffer *(v/v)* (0.5 M Tris-HCl, pH 6.8), 50% glycerol *(v/v)*, 5% SDS *(w/v)*, 3.86% DTT *(w/v)*, a spatula point tip bromophenol blue, H_2_O_ultrapure_ to 10 mL). Samples were incubated at 70 °C for 5 min prior to separation. SDS-polyacrylamide gel electrophoresis (PAGE) was performed according to standard protocols. Proteins were visualized by gel staining with Quick Coomassie Stain (Generon, Slough, UK).

### 2.11. Fluorescence Measurements

Fluorescence measurements were performed with a fluorescence spectrometer (Perkin Elmer LS 50B, PerkinElmer, Waltham, MA, USA). Samples of soluble protein fractions were diluted appropriately (strains of *H. elongata* carrying plasmids pSPE2_GFPuv, pWUB01_GFPuv, and pWUB02_GFPuv 1:500; strains carrying plasmids pSPE1_GFPuv, pPE.GFPuv, and control vector pPromEct 1:50) with H_2_O_ultrapure_. GFPuv (green fluorescent protein variant optimized for maximal fluorescence when excited by ultraviolet light) was excited at 395 nm and the fluorescence emission was recorded as a spectrum between 420–750 nm.

### 2.12. Calculation of Optimized Ribosomal Binding Sites

For the calculation of RBS regions, which are adjusted to the respective expression organism (host), the online tool “RBS Calculator v2.0” was used [27,28]. This tool calculates synthetic RBS regions in such a way as to avoid the formation of secondary structures within the upstream region of the corresponding start codon. As input for “Protein Coding Sequence”, the first 35 bp of the *gfpuv* coding sequence were used. In addition, a pre-sequence was entered, covering 20 bp upstream of the restriction site used for the integration of the construct into the vector (see Table 2). The target translation initiation (t.i.) rate was marked as “maximize”. The input for “organism” depended on the respective vector: for plasmid pSPE1_GFPuv, the last nine nucleotides of the 16S ribosomal RNA (rRNA) of *H. elongata* DSM 2581^T^ (CTCCTTAAT [4], Acc. no. FN869568.1/2) were used. For plasmid pSPE2_GFPuv, *E. coli* K-12 substr. DH10B was chosen from the options of the tool, for which the last nine nucleotides have been annotated as ACCTCCTTA (Acc. no. CP000948.1) [29]. 

## 3. Results

### 3.1. Construction of a Suitable Expression Vector for H. elongata

Genetic modification of an organism is a prerequisite, and a vector based expression of homologous or heterologous proteins a common approach for the exploitation of microorganisms as cell factories. To develop an expression system that is suitable for *H. elongata*, a broad host range vector (pBBR1-MCS) was chosen as a backbone and one of the hosts own promoters was selected. The promoter region upstream of the ectoine biosynthetic gene cluster (*promA*) seemed a good choice as it is active under standard growth conditions (increased salinity) and can be further regulated by increasing or decreasing the salinity of the medium. As this promoter responds to salinity changes, the application and continuous replacement of synthetic unstable inducers, such as IPTG (isopropyl β-d-1-thiogalactopyranoside) or AHT (anhydrotetracycline) becomes dispensible [30].

**Figure 1:**
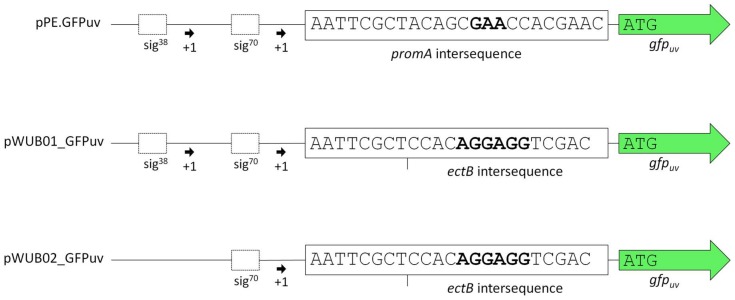
Schematic comparison of the promoter regions in plasmids pPE.GFPuv, pWUB01_GFPuv, and pWUB02_GFPuv. Sequences in bold represent the annotated RBS [4] in the *promA* intersequence of the wild-type and the Shine-Dalgarno consensus sequences upstream of the *ectB* gene.

Vector pPE was constructed by integrating the *promA* promoter region into pBBR1-MCS. Use of an artificial restriction site (PscI, ACATGT) enabled the integration of target genes in place of the *ectA* gene downstream of the promoter region. This was done with *gfp_uv_,* encoding a fluorescent reporter protein. The resulting vector was pPE.GFPuv (see Figure 1, upper lane).

**Figure 2:**
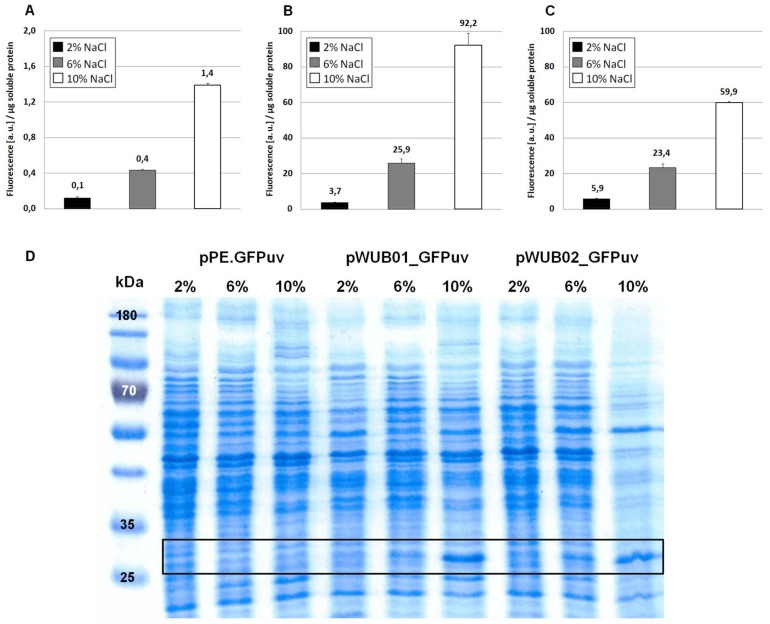
Expression of GFPuv in *H. elongata*, part I. Strains expressing the reporter protein GFPuv were grown in MM63 medium at constant NaCl concentrations (2%, 6%, or 10% *w/v*) until early stationary phase. Then, cultures were harvested and soluble protein fractions were isolated, purified and used for fluorescence measurements and SDS-PAGE. (**A**) *H. elongata* pPE.GFPuv, (**B**) *H. elongata* pWUB01_GFPuv, (**C**) *H. elongata* pWUB02_GFPuv, fluorescence (arbitrary units, a.u.) per µg soluble protein fraction of *H. elongata* strains. (**D**) SDS-PAGE of soluble protein fractions of respective *H. elongata* strains. GFPuv has a size of 26.8 kDa, framed.

As displayed in Figure 2A, the functionality of the construct and its response to changes in medium salinity was proven by production of the reporter protein. However, despite its successful application for several homologous and heterologous protein expressions in *H. elongata* [25,31], the expression levels of vector pPE always remained very low and could not be visualized on a SDS-PAGE (see Figure 2, bottom). A closer look into the ectoine biosynthetic gene cluster revealed that the RBS upstream of the *ectA* gene has hardly any similarity to a perfect Shine-Dalgarno (SD) consensus sequence, while the following *ectB* gene displays such a perfect consensus: AGGAGG with a spacing of five nucleotides. To verify that the weak RBS in the *promA* region upstream of *ectA* leads to weak translation, we constructed the vector pWUB01, which contained the same promoter elements as in pPE, but the RBS from the *ectB* gene (see Figure 1, middle lane). As shown in Figure 2B, GFPuv fluorescence of strains carrying the pWUB01 vector was approx. 50 times higher than that obtained with the pPE vector. The conclusion that the presence of a perfect RBS has a strong impact on translation efficiency was confirmed by strong bands on SDS-PAGE gels, demonstrating a significant increase in the amount of protein. This observation further supported the idea that not the promoter elements (responsible for transcription), but the RBS region (responsible for translation initiation) of the *promA* element is limiting (Figure 2D).

The upstream promoter region of *promA* comprises two elements: σ^38^ and σ^70^. On the basis of the work by Lee & Gralla, 2004 [32] and Rosenthal Interchim Uptima, Montluçon, 2006 [33], the former (GCGG-N13-CTATAAT), despite its suboptimal spacer length, was assumed to be osmoresponsive [4]. We therefore expected that deletion of this region would abolish salt-dependence, and, instead, enable constitutive expression by the σ^70^ element, unaffected by osmotic influences. In vector pWUB02 (Figure 1, bottom lane), the promoter region was shortened to such an extent that only 29 bp remained upstream of the −35 region of the σ^70^ element. This removed the σ^38^ element but left the rest of the sequence unaffected and identical to that of pWUB01. As depicted in Figure 2C, total GFPuv fluorescence was somewhat reduced at higher salinities, but still displayed a strong dependence on NaCl concentration of the medium.

### 3.2. Optimization of Translation Based on Messenger RNA Folding Energies

As described in Section 3.1, a change of a very weak RBS in plasmid pPE to the consensus sequence AGGAGG in pWUB01 resulted in a significant increase in GFPuv expression with plasmid pWUB01_GFPuv (Figure 2B). However, it became apparent from subsequent constructs that thr heterologous protein expression with pWUB01 differed greatly with different target genes from compatible solute synthesizing gene clusters. As the importance of thermodynamic stability of messenger RNA (mRNA) secondary structures for translation efficiency is well established for both the efficient recognition of the start codon by the initiator-transfer RNA (tRNA) [34,35] and the binding of the RBS to the anti-SD sequence at the 16S rRNA, we further investigated the effect of sequence variations within the *promA* region. For this we used a biophysical model, the RBS Calculator v2.0 [27,28]. This tool is based on the Gibbs free energy calculation of mRNA interactions that are involved in ribosome binding and translation and creates a so-called synthetic RBS region (between 20–40 bp in size). Part of this sequence also contains the RBS *sensu stricto* with a spacing of approx. six nucleotides to the start codon. 

**Figure 3:**
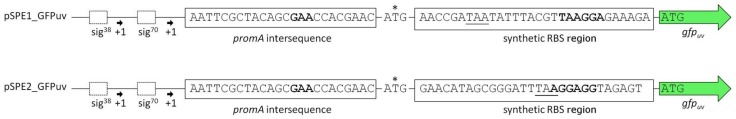
Schematic comparison of the promoter regions in plasmids pSPE1_GFPuv and pSPE2_GFPuv. Sequences in bold are the crucial bases involved in binding of the 16S rRNA as described by Schwibbert et al. [4] and as obtained by the RBS Calculator v2.0 [27,28]. The original start codon ATG (*) has little relevance in this context because of its weak RBS (GAA) and an in-frame stop codon further downstream (underlined).

Analysis of the annotated 16S rRNA of *H. elongata* DSM 2581^T^ [4] revealed that it differed from the expected consensus of other gamma-proteobacteria in its 3′ end (see discussion). Therefore, we also chose the consensus sequence of the *E. coli* 16S rRNA as a reference and comparison. The resulting two plasmids pSPE1_GFPuv (RBS adapted to 16S rRNA sequence of *H. elongata* DSM 2581^T^) and pSPE2_GFPuv (RBS adapted to the consensus sequence present in *E. coli*) are displayed in Figure 3. They were created from the pPE vector with both promoter elements, but contain an additional sequence representing a synthetic RBS region followed by *gfpuv*. Table 3 shows the predicted translation initiation rates (t.i. rates) of the two genetic constructs, as obtained from the RBS Calculator v2.0. t.i. rates are given as arbitrary units and represent the relative translation rate of a protein coding sequence with a maximum for *E. coli* K12 of 5,687,190 arbitrary units (a.u.) [36]. From this one would expect a stronger translation of pSPE1_GFPuv in *H. elongata,* reflecting the optimization for the last nine nucleotides of its 16S rRNA.

**Figure 4:**
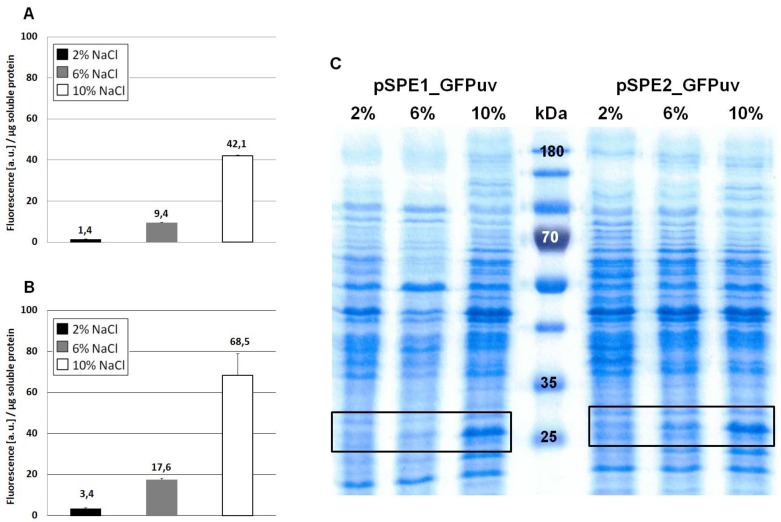
Expression of GFPuv in *H. elongata*, part II. Strains expressing the reporter protein GFPuv were grown in MM63 medium at constant NaCl concentrations (2%, 6% or 10% *w/v*) until early stationary phase. Then, cultures were harvested and soluble protein fractions were isolated, purified and used for fluorescence measurements and SDS-PAGE. (**A**) *H. elongata* pSPE1_GFPuv, (**B**) *H. elongata* pSPE2_GFPuv, fluorescence (a.u.) per µg soluble protein fraction of *H. elongata* strains. (**C**) SDS-PAGE of soluble protein fractions of respective *H. elongata* strains. GFPuv has a size of 26.8 kDa, framed.

When comparing Figure 2A (no optimization) with Figure 4A (sequence optimization according to RBS Calculator) it becomes obvious that GFPuv expression with the pSPE1_GFPuv vector was significantly stronger than the original pPE.GFPuv vector. A 30-fold increase in fluorescence of cultures grown at 10% NaCl clearly proved the usefulness of a sequence optimization process with respect to molecular interactions important for translation initiation. Against expectations, however, GFPuv production was even higher with plasmid pSPE2_GFPuv (Figure 4B), although its synthetic RBS region was optimized for the expression in an organism possessing a 16S rRNA 3′ end sequence identical to that of *E. coli*, rather than the one proposed for *H. elongata* DSM 2581^T^.

In this case, an almost 50-fold increase can be noticed (growth at 10% NaCl) when compared to Figure 2A (no optimization). The overall fluorescence intensity that is gained by expression with plasmid pSPE2_GFPuv is close to the results that were obtained from pWUB01_GFPuv and pWUB02_GFPuv (Figure 4B and Figure 2B,C), both of which contain an SD consensus sequence. SDS-PAGE gels support these fluorescence data, clearly documenting an increased production of GFPuv with increasing NaCl concentration of the medium (Figure 4C). 

### 3.3. Periplasmatic Expression of GFPuv and mCherry

Having successfully established cytoplasmatic over-expression of proteins in a moderately halophilic host, we became interested in the possibility to export the expression products into the periplasm. *H. elongata*—as a moderate halophile—offers two distinct milieus simultaneously. On the one hand, a cytoplasm filled with folding aids (compatible solutes), and on the other hand a highly ionic periplasmatic space, which might be beneficial for the folding and stability of strictly halophilic proteins, provided that they can be exported. Therefore, we investigated leader sequences belonging to the twin-arginine translocation (Tat) system (transport of folded proteins) and the Sec translocon (unfolded transport). First, we identified *tat* and *sec* genes in the genome of *H. elongata* DSM 2581^T^ [4]. The Copper resistance protein PcoA (HELO_1864) made a promising candidate with congruent identification of a Tat leader peptide by PRED-TAT [37] and TatP1.0 [38]: MSMPQKPLLPLTRRQLLKGGSALGISTMALGLPPAWA-SPWG (twin-arginin motif and cleavage site underlined). Already well investigated is the periplasmatic ectoine binding protein TeaA (HELO_4274) as part of the solute transport system TeaABC [17], which contains in its unprocessed form a Sec signal peptide also identified by SignalP-4.0 [39]: MKAYKLLTTASIGALMLGMSTAAYS-DNWR (cleavage site underlined). DNA fragments encoding these leader peptides plus four residues of the corresponding mature proteins were fused in frame to *gfpuv* (Tat) or *mCherry* (Sec), respectively. The resulting constructs were cloned into the pWUB01 vector and transferred into *H. elongata.*

**Figure 5:**
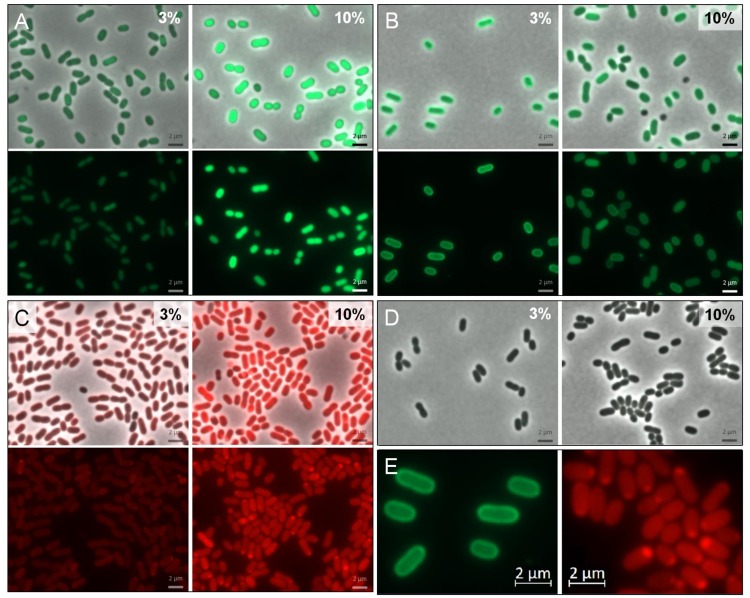
Imaging of cytoplasmatic and periplasmatic expression of GFPuv and mCherry in *H. elongata*. Cells were either grown at constant salinity in MM63-3% or grown in MM63-3% and shocked to 10% NaCl at an OD_600nm_ of 0.4–0.5 with continued growth for 4 h. Then, cells were harvested and used for fluorescence microscopy. In groups A–C, the upper frame shows the merge channel, the lower frame only the respective color channel (either for GFPuv or mCherry). (**A**) *H. elongata* pWUB01_GFPuv (control, cytoplasmic expression) (**B**) *H. elongata* pWUB01_tat_GFPuv (**C**) *H. elongata* pWUB01_sec_mCherry (**D**) *H. elongata* pWUB01 (control, empty vector) (**E**) Magnification of observed phenotype; left: typical periplasmic “halo” of *H. elongata* pWUB01_tat_GFPuv; right: polar protein accumulation in *H. elongata* pWUB01_sec_mCherry.

Comparison of cytoplasmic and periplasmic GFPuv expression clearly shows a fluorescent “halo” in respective cells at 3% NaCl (Figure 5B,E, left). This is an indication for the successful transport of GFPuv in an active folded state into the periplasm, mediated by the selected Tat leader peptide. At 10% salinity this effect (although still visible) appears to be less pronounced and it may be due to a congestion of the export system. In cells expressing mCherry, the expected “halo” is not very pronounced. Instead, a polar accumulation of fluorescent proteins can be observed, especially under high salt conditions. 

## 4. Discussion

### 4.1. Characterization of the Promoter Region Upstream of ectA

As the genetic organization of ectoine biosynthetic genes has been studied in detail [4,5,6,7], we expected the corresponding promoter region upstream of *ectA* (*promA*) to be well suited for the construction of an expression vector in *H. elongata.* Schwibbert et al., 2011 have analyzed the *H. elongata* DSM 2581^T^ promoter region 132 bp upstream of *ectA* and concluded a putative set of one σ^38^ (GCGG—N13—CTATAAT) promoter, followed by a standard σ^70^ element (TTGAAA—N17—TATGAT), the former being regarded as salt-responsive. This conclusion was drawn from the presence of a so-called G-element (consensus GCGG) at position −35, which was found to be a conserved element in six osmotically induced σ^38^ promoters of *E. coli* [32]. Our results confirmed that the *promA* region indeed enables a salt-dependent expression (Figure 2A,B), which according to literature should be caused by the σ^38^ promoter element. It therefore seemed likely that this salt-dependent expression system could be converted into a constitutive one by removing the σ^38^ element so that only the vegetative σ^70^ promoter remained. Surprisingly, construction of vector pWUB02—which lacks the σ^38^ element—did not lead to constitutive expression of GFPuv. Instead, it remained strongly salt-dependent. In fact, the results gained from fluorescence measurements were very similar to vector pWUB01 and suggested that it made little difference, whether both sigma elements were available or just σ^70^ (see Figure 2B,C). In another moderate halophile, *Chromohalobacter salexigens*, the ectoine gene cluster was shown to be controlled by four promoters [40], again σ^70^ and σ^38^ type. Corroborating our own findings in *H. elongata,* one of the σ^38^ elements contained a G-element, but was actually the only promoter element not to be induced by salt. The other three promoters were osmoregulated, but in a follow-up study, the authors proved that σ^38^ is not needed for osmotic regulation of the ectoine gene transcription after all [41]. These findings contradict the need of a G-element (GCGG) for salt-dependent expression. Even in *E. coli*, the role of the G-element is recently discussed to be questionable as it was deleted without a relevant effect [33,42]. Deviating somewhat from the work on *E. coli* [32], where the G-element was found to be separated by a 15 bp spacer from the −10 element, this region in *H. elongata* DSM 2581^T^ comprises only 13 bp. Furthermore, Zhu et al., 2014 confirmed this pattern of a G-element in only one (*Halomonas* sp. QHL1, spacing of 16 bp) out of four related species [43]. If or if not σ^38^ is involved in ectoine gene transcription in *H. elongata* at all remains unclear, as the corresponding promoter region does not fit in all the aspects the expected consensus sequence [44]. It is however justified to conclude that the σ^70^ promoter region alone is sufficient to mediate salt-dependency (Figure 2). This is also supported in a recent study by Czech et al., 2017 [45], who have successively truncated the 230 bp upstream region of the ectoine gene cluster of *Pseudomonas stutzeri* A1501 and demonstrated, using *ect-lacZ* reporter fusions, that the σ^70^ promoter region closest to *ectA* is sufficient to establish a salt-related response—even when expressed in *E. coli*. As the −35 (TTGAGA) and −10 (TACCCT) sequence deviated considerably from that of a perfect σ^70^ consensus, the authors have subsequently mutated this region towards a perfect sequence (TTGACA—N18—TATAAT) and observed a dramatic increase in basic and salt-induced protein production. The important point is that even a consensus σ^70^ promoter proved to respond to increased salinity when expressing *lacZ* as a reporter gene. As Czech et al. observed this effect in a heterologous expression system in *E. coli*, the salt response of a constitutive ectoine promoter cannot depend only on regulatory mechanisms of a natural ectoine producer strain. Still, one has to bear in mind that the genetic boundaries of the promoter region may also play a role and that salt-response may at least partially occur at the level of translation. 

### 4.2. Optimizing Translation Initiation Using the RBS Calculator v2.0

With vector pPE (carrying the natural *promA* region), we were able to show that the protein expression controlled by external NaCl concentration is possible in *H. elongata* [25,31], but the overall expression level fell short of our expectations. A closer look into the *promA* region revealed that the sequence upstream of *ectA* has little similarity with a ribosomal binding site, although a GAA motif 8 bp upstream of the start codon was suggested by Schwibbert et al. [4]. Lack of a RBS is not unusual in bacterial genomes, in fact, approximately 15% of the genes from gamma-proteobacteria do not have one [46]. Still, we had to note that the subsequent genes *ectB* and *ectC* are equipped with SD motifs (AGGAGG and GGAG, respectively), which are the most commonly used in the prokaryotic world [46]. As we pursued the concept of a highly efficient heterologous protein production system, including highly effective translation, we tested the hypothesis that the pPE vector—or rather the *promA* promoter—was just in need of a stronger ribosomal binding site. Fortunately, such a situation was found in an ectoine deletion mutant (*H. elongata* KB1 Δ*ectA*), where the *promA* region is fused to the perfect SD motif AGGAGG upstream of *ectB* [17]. The resulting vector pWUB01 indeed accomplished strong over-expression of our reporter gene (Figure 2B), supporting our suspicion that not transcription, but translation initiation was the limiting factor in the vector-based *promA* system.

While GFP expression reliably yielded very good results with the pWUB01 vector and in addition a selection of other proteins was successfully over-expressed in our laboratory in *H. elongata*, we occasionally failed to reproduce perfect performance for some other investigated proteins. A subsequent more systematic approach employed the RBS Calculator v2.0 [27,28]. This bioinformatic tool adapts a RBS region to the host and the respective target gene. It requires the last nine nucleotides of the host’s 16S rRNA as input for its calculations, as this 3′ terminus usually comprises the anti-SD-motif, which is complementary to the RBS sequence in the mRNA, and, therefore, mediates the ribosomal binding. Comparison of the last 20 nucleotides of the annotated 16S rRNA of *H. elongata* DSM 2581^T^ [4] with *E. coli* K-12 substr. DH10B as an example for a consensus sequence [47] revealed a strong similarity, the only difference being an AT extension in the case of *H. elongata.* (*H. elongata* DSM 2581^T^: 5′-GGCTGGATCACCTCCTTAAT-3′; *E. coli* K-12 substr. DH10B: 5′-GCGGTTGGATCACCTCCTTA-3′). However, as the CCTCCT sequence (complementary sequence to AGGAGG) was placed further upstream according to its annotation, it would not be recognized as a perfect consensus for a calculation by the RBS Calculator. Our comparison of a synthetic RBS region designed for the annotated sequence of *H. elongata* (pSPE1) with another designed for *E. coli* with the perfect anti-SD motif (pSPE2) (Figure 3) confirmed our suspicion that the annotation of 16S rRNAs of *H. elongata* may be incorrect. Such flaws in genome annotation are not unusual [48], and we therefore contacted the authors on this matter, which led to a recent correction of the previous annotation [49]. It has now been clarified that the last 15 nt of the 16S rRNA of *H. elongata* DSM 2581^T^ correspond 100% with those of *E. coli* K-12 substr. DH10B (consensus sequence 5′-TGGATCACCTCCTTA-3′). This consensus was found to be highly conserved in a number of different species originating from different phyla [47]. By way of clarifying the 3′ terminus of its 16S rRNA and demonstrating the usefulness of the RBS Calculator experimentally, we have now paved the way for successful over-expression of any target protein in this halophilic host.

### 4.3. Directing Proteins into the Periplasmic Environment

*H. elongata* DSM 2581^T^ offers an interesting combination of environments for proteins: the cytoplasm with an osmotic equilibrium that was created by compatible solutes, like ectoine and an ionic periplasm, which can act as a mediator between potentially high salt media and the cytoplasm. Having established cytoplasmatic expression of proteins in *H. elongata,* as shown above, we looked for suitable signal peptides for directed transport into the periplasm. With PcoA (HELO_1864) and TeaA (HELO_4274), we found two signal peptides that were appropriate for a Tat or a Sec mediated transport. GFPuv is known to remain inactive when transported into the periplasm in an unfolded state via Sec pathway [50,51], therefore this reporter protein is well suited to investigate the Tat export pathway. In contrast, the fluorescent protein mCherry can be exported via Sec pathway to the periplasmatic space, where it subsequently folds into the active state [51].

In case of Tat mediated export, we were able to show that the chosen leader sequence was suitable for directed transport of functionally folded GFPuv in *H. elongata* (Figure 5). Nevertheless, we observed a decrease in the translocated GFPuv when growing *H. elongata* strains in medium containing 10% NaCl (Figure 5B), which coincides with high expression levels. Higher expression (and therefore fluorescence) triggered by an increased NaCl concentration is bound to impede a clear visual distinction between periplasm and cytoplasm. As a result, only a weak “halo” is recognizable. We assume that this is also caused by capacity overload of the TatABC translocase. This observation has been made before in thylakoid membranes and gram negative bacteria [52,53,54] and is described as a saturation of the Tat system when proteins are overproduced. Due to this overload, overproduced Tat-GFPuv might accumulate in a precursor form in the cytoplasm, unable to fold a fluorescent complex [55]. 

In the case of mCherry translocation via Sec pathway, we observed a rather uneven distribution with strong fluorescence being localized at the cell poles especially at high salt concentration (Figure 5C,E, right). Such an observation has also been made before in *E. coli* with both Sec-translocated fluorescent protein [51] and Tat leader sequences [56]. Reorganization and compartmentalization of the periplasm following osmotic upshocks has been proposed as a possible explanation for such observations. We cannot—at present—convincingly conclude that Sec transport of mCherry has been successful and must admit the preliminary nature of these observations. Convincing proof would require the isolation of the proteins through cell fractionation experiments. We have tried to apply standard protocols as described for *E. coli* [57], but have so far failed to obtain a periplasmic fraction of our halophilic model organism. However, the promising results with GFPuv translocation encourage further work addressing protein export into the periplasm by either Sec or Tat transport systems, since it opens up the unique option to combine heterologous protein production with a drastic change of the environment from solute to salt.

## 5. Conclusions

With this work, we established a salt-dependent, compatible solute supported, cytoplasmatic, and periplasmatic expression system in the moderate halophile *H. elongata* DSM 2581^T^. Further projects may target the development of a more constitutive promoter system to uncouple protein expression from the salt-regulated production of compatible solutes. Recombinant protein expression in *H. elongata* seems to be a promising strategy for labile products in need of a stabilizing environment. The cytoplasm offers folding aids (compatible solutes), while the periplasm offers the additional advantage of exposing the product to high ionic strength, as required for the correct folding of halophilic proteins. We are now in a position to not only optimize heterologous protein expression in *H. elongata* using the RBS Calculator, but also to target both cell compartments. Future work will address the benefit of this expression system for biotechnological applications.

## Figures and Tables

**Table 1:** Bacterial strains and plasmids used in this work.

	Relevant Characteristics/Description	Source/Reference
**Strains**		
*Halomonas elongata*		
DSM 2581^T^	wild type	[1]
KB1	Δ*ectA*	[17]
*Escherichia coli*		
S17.1	RP4-2 (Tc::Mu) (Km::Tn7); Sm^R^, *pro, thi, recA*	[19]
**Plasmids**		
pBBR1-MCS	Broad host range cloning vector	[22,23]
pGFPuv	Expression vector containing the gene sequence of GFPuv	Clontech Laboratories, Inc. (Mountain View, CA, USA)
pCQ11-ftsZmCh	Contains the gene sequence of mCherry, a DsRed derivative	[24]
pPE	Cloning vector; pBBR1MCS derivative equipped with the salt-dependent *H. elongata* promoter region approx. 470 bp upstream of *ectA* (HELO_2588)	[25,26]
pPE.GFPuv	pBBR1MCS derivative equipped with the promoter region upstream of *ectA* (HELO_2588)*,* followed by *gfpuv*	[25]
pSPE1_GFPuv	pBBR1MCS derivative equipped with the promoter region upstream of *ectA* (HELO_2588), followed by a synthetic RBS region optimized for *H. elongata* DSM 2581^T^ and the gene *gfpuv*	This work
pSPE2_GFPuv	pBBR1MCS derivative equipped with the promoter region upstream of *ectA* (HELO_2588), followed by a synthetic RBS region optimized for *E. coli* K-12 substr. DH10B and the gene *gfpuv*	This work
pWUB01_GFPuv	pBBR1MCS derivative equipped with the promoter region upstream of *ectA* (HELO_2588) from *H. elongata* DSM 2581^T^ and the RBS upstream of *ectB* (HELO_2589) from *H. elongata* KB1 (*promKB1*), followed by *gfpuv*	This work
pWUB02_GFPuv	pBBR1MCS derivative equipped with part of the promoter region upstream of *ectA* (HELO_2588) (including only the σ^70^ sequence) from *H. elongata* DSM 2581^T^ and the RBS upstream of *ectB* (HELO_2589) from *H. elongata* KB1 (*promKB1*), followed by *gfpuv*	This work

GFPuv: green fluorescent protein variant optimized for maximal fluorescence when excited by ultraviolet (UV) light; RBS: ribosome binding site; bp: base pairs.

**Table 2:** Primers used for vector cloning. Sequences in bold are synthetic RBS regions calculated with the RBS Calculator [27,28]. Underlined sequences refer to restriction sites.

Primer	Sequence (5′ → 3′)	Restriction Site
for_1A	GGAGGCCGTCTAGATCATCCAGG	XbaI
rev_1A	CTCTGTGGATCCGTACATGTTCGTGGT	BamHI, PscI
rev_2B	AATGGATCCCTACATGTCGACCTCCTGT	BamHI
for_2C	TATTCTAGAGGAATTCAGCAAGCAAGAT	XbaI
rev_3C	ATAACAATTTCACACAGGAAACAGCTA	-
for_3D	CCGGTAGAAATCATGAGTAAAGGAGAAG	PagI
rev_4D	GGCCGACTAGTAAGCTTATTATTTTTGACAC	HindIII
for_4E	ATTCTGCAG**AACCGATAATATTTACGTTAAGGAGAAAGA**ATGAGTAAAGGAGAAGAACTT	PstI
for_5E	ATTCTGCAG**GAACATAGCGGGATTTAAGGAGGTAGAGT**ATGAGTAAAGGAGAAGAACTT	PstI
for_6F	ATTTCTAGAGGGCGCGAAGCCTGCCCGTC	XbaI
rev_5F	ATTGGATCCTTGCAATCTTCCTTATGACT	BamHI
for_7F	ATTAAGCTTGAACATAGCGGGATTTAAGG	HindIII
for_8G	CAAGCAAGCCGAACTGGACGCCGAACGC	-
for_9G	ACAATCATGAAGGCATACAAGCTGCTGAC	PagI
rev_6G	TCGCCCTTGCTCACACGCCAGTTGTCGG	-
for_10G	CCGACAACTGGCGTGTGAGCAAGGGCGA	-
rev_7G	ATTAAGCTTATAGGCGCGCCTTACTT	HindIII
for_11H	TTCGATCCTGATCCAGTTGCTTGATCA	-
for_12H	ATTACATGTCAATGCCCCAGAAGCCTTTA	PscI
rev_8H	TTCTTCTCCTTTACTTCCCCAGGGACTGG	-
for_13H	CCAGTCCCTGGGGAAGTAAAGGAGAAGAA	-

**Table 3:** Comparison of predicted translation initiation rates (a.u.) calculated with the RBS Calculator v2.0. The calculation is based on the respective gene (*gfpuv*) and the last nine nucleotides of the annotated 16S rRNA of the respective organism.

Vector	*H. elongata* DSM 2581^T^	*E. coli* DH10B
pSPE1_GFPuv	365,234.33	65,308.16
pSPE2_GFPuv	95,871.18	1,231,096.69
